# Axel W. Bauer, Ralf-Dieter Hofheinz, Jochen S. Utikal (Hrsg) (2021) Ethical Challenges in Cancer Diagnosis and Therapy: Series: Recent Results in Cancer Research, 218, Springer, Cham, 290 Seiten, 139,09 €, ISBN ISBN 978-3-030-63748-4

**DOI:** 10.1007/s00481-022-00685-y

**Published:** 2022-02-01

**Authors:** Eva C. Winkler

**Affiliations:** Sektion für Translationale Medizinethik, Nationales Centrum für Tumorerkrankungen (NCT), Medizinische Onkologie, Universitätsklinikum Heidelberg, Heidelberg, Deutschland

Dieses Buch ist ein Sammelband mit 18 Kapiteln zu ganz unterschiedlichen Themen, die im weitesten Sinne relevant werden können im Kontext der Diagnostik und Therapie von Krebspatient:Innen. Viele Themen sind jedoch sicher nicht spezifisch für die Onkologie wie etwa das Recht auf Nichtwissen in der genetischen Prädiktion (Kap. 6), die regulatorischen Überlegungen zur Medikamentenzulassung der EMA oder das Thema Vorausverfügungen.

Während andere Sammelbände zu dem Thema beispielsweise aus Konferenzbeiträgen zusammengestellt sind, gibt das Editorial leider keine Auskunft zum Hintergrund und Ziel dieses Sammelbandes. Damit bleibt auch etwas unklar, nach welchen Kriterien die Auswahl der Themen und Autor:Innen erfolgt ist, denn die Autor:Innen und damit auch die Beiträge kommen aus ganz unterschiedlichen Fachdisziplinen: überwiegend aus den Lebenswissenschaften, teilweise aus Geschichte, Theorie und Ethik in der Medizin, aus dem Recht und aus den Sozial- und Literaturwissenschaften. Da sich mir der thematische Aufbau nicht erschlossen hat, versuche ich hier für zukünftige Leser:Innen die 18 Kapitel thematisch gruppiert vorzustellen und einzuordnen.

Im ersten Kapitel demontiert Eckart in einer kritischen Auseinandersetzung mit dem populären Buch von Murkajee das verbreitete Narrativ des „Cancer as Emperor of all maladies“ mit profundem medizinhistorischen Wissen. Er betont, dass Krebs nur für die westlichen und wohlhabenden Länder, die die Infektionskrankheiten gut behandeln können, einen so hohen Stellenwert hat. Mit Blick auf die globale Krankheitslast der Menschheit waren es – auch schon vor der Covid 19 Pandemie – immer die Infektionskrankheiten, die dem Menschen am gefährlichsten wurden. Hier lassen sich interessante Bezüge zum Editorial herstellen, in dem die Sorge deutlich wird, dass durch die Covid 19 Pandemie der Krebsforschung weniger Forschungsfördermittel zukommen, wenn die Infektionsforschung offensichtlich an Bedeutung gewinnt.

Vier Kapitel (Nr. 2, 5–7) befassen sich mit Themen der Prädiktion und Prävention in der Medizin. Kapitel 2 und 7 erklären die statistischen Fallen bei der Effektmessung der Krebsvorsorge. Während es Weiss (Kap. 2) als Biostatistikerin vor allem ein Anliegen scheint, mit der Annahme aufzuräumen, dass Krebsvorsorge Leben rettet, und vor den Gefahren von Überdiagnostik und fehlendem statistischen Verständnisses bei vielen Ärzten, Journalisten und Gesundheitsexperten zu warnen, bestätigt Robra (Kap. 7) als Gesundheitswissenschaftler diesen Befund, geht aber weit darüber hinaus: Er zeigt zum einen an der derzeitigen Studienlandschaft, welche Qualitätskriterien und Evidenzlevel erfüllt sein müssen, um Entscheidungsträger in die Lage zu versetzten, Nutzen und Schaden von Vorsorgeprogrammen abzuwägen. Während diese Entscheidungen notwendigerweise einem utilitaristischen Nutzenkalkül folgen, zeigt er darüber hinaus die Möglichkeiten und Lücken auf dem Weg zur gut informierten Entscheidung des Einzelnen mit Blick auf Vorsorgeuntersuchungen auf. Ein Kapitel mit reichlich Anknüpfungspotential für die Public Health Ethik.

Die medizinischen, ethischen und vor allem rechtlichen Herausforderungen der prädiktiven Medizin werden von der Arbeitsgruppe Schmutzler, Dabrock, Huster vorgestellt. Dieser Beitrag hebt sich insofern von vielen anderen des Sammelbandes ab, weil er die ethische Frage konkret benennt und hier vor allem auch rechtliche Analyse und Lösungsansätze umfasst. Hier geht es um die Frage, ob eine prophylaktische Mastektomie bei Menschen mit hohem erblichen Brustkrebsrisiko in den Leistungskatalog der Krankenkassen aufgenommen und damit erstattet werden soll. Der Beitrag berichtet damit die Studienergebnisse des BMBF-geförderten Syskon-Konsortiums und hebt sich auch damit ab, dass hier aktuelle Studienergebnisse und aktuelle Literatur aus Deutschland berichtet werden. In einem weiteren Kapitel beleuchtet Duttge die Entwicklung und Bedeutung des Rechts auf Nichtwissen aus juristischer und gesellschaftlicher Sicht.

Einen sehr guten Überblick über forschungsethische Fragen aus regulatorischer Sicht gibt das Autorenteam Guizzaro (EMA) und Kihlborn (Forschungsethik Uppsala). Von der Rechtfertigung einer (oft auch kritisierten) aufwändigen Forschungsaufsicht, über Tierversuchsethik bis zu den methodischen Herausforderungen, der Heterogenität von Patientenpräferenzen bei der Medikamentenentwicklung gerecht zu werden, wird der Diskussionstand zu wichtigen regulatorisch-ethischen Fragen gut verständlich zusammengefasst. Ein zweiter Beitrag greift das forschungsethisch bekannte Thema der HELA-Zellkulturen auf, das sonst vor allem mit Blick auf potenzielle urheberrechtliche Ansprüche diskutiert wird. Hier werden interessanterweise vor allem Fragen der Qualitätssicherung von Zellkulturen aus mikrobiologischer Sicht aufgegriffen, während die ethische Debatte kursorisch und bestenfalls verkürzt widergegeben wird.

Eine weitere Gruppe von Kapiteln (10–14) befasst sich mit Entscheidungen und Begleitung von Menschen am Lebensende. Feldmann und Benrath beschreiben die Begleitung der letzten Lebensphase aus schmerztherapeutisch und psychotraumatologischer Sicht – sowohl dass statt von Patienten von „Klienten“ die Rede ist als auch die etwas esoterisch anmutende Transition zu Shangri-La und mentalen Energiefeldern lassen die Rezensentin zwar etwas ratlos zurück – zugleich beschreiben die Autoren aber die Wünsche und die Bedeutung der rechtzeitigen und kontinuierlichen (Gesprächs‑)Begleitung sehr gut. Sehr viel nüchterner und strukturiert fasst Beckmann die Funktion und den rechtlichen Rahmen von Vorausverfügungen für medizinische Entscheidungen am Lebensende im Allgemeinen zusammen. In der Onkologie sind jedoch die meisten Patienten und Patientinnen bis zum Lebensende sehr wohl kommunikationsfähig, so dass sich hier die Diskussion um Entscheidungen am Lebensende wesentlich in Richtung vorausschauende Behandlungsplanung (advance care planning) verlagert hat. Auch der Beitrag von Bauer zum ärztlich assistierten Suizid lässt den Bezug zur Onkologie und den mittlerweile wiederholt erhobenen empirischen Daten zu dem eher seltenen Phänomen tatsächlicher Inanspruchnahme assistierter Suizidmöglichkeiten unter Krebspatienten vermissen. Der Schwerpunkt liegt vielmehr auf der von Bauer wiederholt geäußerten Kritik an der Medizinethik, die sich zum willfährigen Gehilfen einer „Thanatopolitik“ machen lässt, hinter der sinistre soziale und ökonomische Interessen stehen, die unter dem Deckmantel der Selbstbestimmung ein Frühableben unproduktiver Bürger erleichtern wollen.

Zwei weitere Kapitel heben sich in ihrer gleichermaßen kenntnisreichen Beschreibung des spezifisch onkologischen Handlungsfelds wie der dazu gehörigen ethischen Debatte hervor – eines zu den ethischen Fragen der pädiatrischen Onkologie (Benedetti & Marron) und ein kritischer Beitrag zur Rolle der Medien und Pressestellen forschender akademischer Institutionen am Beispiel des Hypes um den Einsatz von Methadon zur Krebstherapie (Steger & Schochow). Die letzten Kapitel stellen die Krankheitserfahrung und speziell die von Krebserkrankungen heraus – durch ein Patiententagebuch und einen sehr interessanten Aufsatz zur Darstellung von Krebs in der Literatur (Engelhardt). Sie erweitern damit den Blick auf das, was Endlichkeit und Krankheitserfahrung für den Einzelnen bedeutet und wie diese Wahrnehmung einerseits kulturell geprägt und zum anderen in Kunst und Literatur verarbeitet wird. Wie viele andere Kapitel werfen sie dabei keine spezifischen ethischen Fragen im Sinne des Buchtitels auf.

Wertvoll aus medizinethischer Sicht sind gerade die Berichte der Kliniker aus ihrer Behandlungspraxis wie der Beitrag zur Lebendspende von der Autorengruppe um Nadalin oder der Beitrag von Hofheinz zur Belastung und Burnoutrisiko von Onkolog:Innen in einem ökonomisierten Gesundheitssystem, das gerade keinen Raum lässt, der menschlichen Herausforderung gerecht zu werden, die eine Krebserkrankung sein kann. In diesen Berichten aus den klinischen Handlungsfeldern wird zwar auf die bestehenden medizinethischen Diskurse nicht Bezug genommen, sie sind aber gute Ausgangspunkte für einen solche Diskurs. Neben den wenigen Kapiteln mit explizitem Bezug auf den ethischen Diskurs sehe ich hierin den Mehrwert dieser Aufsatzsammlung im Sinne der praktischen Ethik: Die Themen werden aus der Sicht der Experten und Expertinnen in ihrem jeweiligen Fachgebiet dargestellt und sind für interessierte Ethiker:Innen ein guter Einstieg und Ausgangspunkt für eine Untersuchung der relevanten ethischen Fragen und möglicherweise erster Antworten auf diese Fragen.

